# *Plasmodium falciparum* coinfection is associated with improved IgE and IgG3 response against hookworm antigens

**DOI:** 10.1002/hsr2.672

**Published:** 2022-06-14

**Authors:** Samuel A. Sakyi, Michael D. Wilson, Bright Adu, Stephen Opoku, Antwi Brewoo, Amma Larbi, Emmanuel K. Baafour, Samuel K. Tchum, Roland O. Saahene, Wilfred Aniagyei, Christian Sewor, David Courtin, Michael Cappello, Ben Gyan, Benjamin Amoani

**Affiliations:** ^1^ Department of Molecular Medicine, School of Medical Sciences Kwame Nkrumah University of Science and Technology Kumasi Ghana; ^2^ Parasitology Department, Noguchi Memorial Institute for Medical Research College of Health Sciences, University of Ghana Legon Ghana; ^3^ Department of Immunology, Noguchi Memorial Institute for Medical Research College of Health Sciences, University of Ghana Legon Ghana; ^4^ Kumasi Center for Collaborative Research in Tropical Medicine Kumasi Ghana; ^5^ Department of Microbiology and Immunology, School of Medical Sciences University of Cape Coast Cape Ghana; ^6^ Department of Biochemistry and Biotechnology Kwame Nkrumah University of Science and Technology Kumasi Ghana; ^7^ Kintampo Health Research Center, Ghana Health Service Kintampo‐North Ghana; ^8^ Department of Microbiology and Immunology, School of Medical Sciences University of Cape Coast Cape Ghana; ^9^ Department of Biomedical Sciences, School of Allied Health Sciences University of Cape Coast Cape Coast Ghana; ^10^ UMR 261 MERIT Institut de Recherche pour le Développement (IRD), Université de Paris Paris France; ^11^ Partnerships for Global Health, Department of Pediatrics, Yale School of Medicine Yale University New Haven Connecticut USA

**Keywords:** coinfection, hookworm infection, immune response, malaria parasite, parasite antigens

## Abstract

**Background:**

*Plasmodium falciparum* and Hookworm infections are prevalent in West Africa and they cause iron deficiency anemia and protein malnutrition in Children. Immune response of these parasites interact and their interactions could have repercussions on vaccine development and efficacy. The current goal of hookworm eradication lies on vaccination. We evaluated the effect of *P. falciparum* coinfection and albendazole treatment on naturally acquired antibody profile against hookworm L3 stage larvae antigen.

**Methods:**

In a longitudinal study, 40 individuals infected with *Necator americanus* only, 63 participants infected with *N. americanus* and *P. falciparum*, and 36 nonendemic controls (NECs) were recruited. The study was done in the Kintampo North Metropolis of Ghana. Stool and blood samples were taken for laboratory analyses. Serum samples were obtained before hookworm treatment and 3 weeks after treatment.

**Results:**

The malaria‐hookworm (*N. americanus* and *P. falciparum*) coinfected subjects had significantly higher levels of IgE (*β* = 0.30, 95% CI = [0.12, 0.48], *p* = 0.023) and IgG3 (*β* = 0.15, 95% CI = [0.02, 0.52], *p* = 0.004) compared to those infected with hookworm only (*N. americanus*). The *N. americanus* groups had significantly higher levels of IgG3 (*β* = 0.39, 95% CI = [0.14–0.62], *p* = 0.002) compared to the control group. Similarly, *N. americanus* and *P. falciparum* coinfected participants had significantly higher levels of IgE (*β* = 0.35, 95% CI = [0.70–0.39], *p* = 0.002) and IgG3 (*β* = 0.54, 95% CI = [0.22–0.76], *p* = 0.002). Moreover, albendazole treatment led to a significant reduction in IgE, IgA, IgM, and IgG3 antibodies against hookworm L3 stage larvae (*p* < 0.05)

**Conclusion:**

*P. falciparum* is associated with improved IgE and IgG response against hookworm L3 stage larvae. Treatment with single dose of albendazole led to reduction in naturally acquired immune response against hookworm infection. Thus, *P. falciparum* infection may have a boosting effect on hookworm vaccine effectiveness.

## 1 INTRODUCTION

Hookworm infection commonly caused by *Ancylostoma duodenale* and *Necator americanus*, is an endemic parasitic disease that affects about five hundred million people in humid regions of Africa, Asia, and South America.^1^ The adult hookworms can persist for years in the host gastrointestinal tract, triggering devastating effects such as iron deficiency anemia and protein malnutrition, particularly in children.^2^ The highest malaria incidence and mortality occur in humid and subtropical regions of Africa,^3^ where *Plasmodium falciparum* parasites are the most prevalent malaria species.^4^ This coinciding geographical circulation of hookworm and *P. falciparum* leads to frequent coinfections.^5^

Infection with *P. falciparum* and hookworm activate altered immune responses. Hookworm elicits a significant immune polarization toward T‐helper‐2 (Th2),^6^ characterized by higher amounts of inflammatory cytokines including interleukin‐5 (IL‐5), IL‐4, IL‐13, and increased levels of immunoglobulin E (IgE).^7^, ^8^ In spite of these robust Th‐2 responses, adult larvae mostly endure in their host for years. The longstanding persistence of the helminths within a seemingly immunocompetent subjects is expedited by the initiation of immunomodulatory mechanisms such as stimulation of T regulatory cells and modulation of cells of the immune system, including dendritic cells and macrophages which results, in an anti‐inflammatory milieu, characterized by high levels of TGF‐β.^9^, ^10^ However, the immune reaction in coinfected *P. falciparum* participants may alter the naturally acquired immune response.^11^ Contrary to hookworm infection, *P. falciparum* infection generates a Th1‐type immune response, resulting in higher levels of TNF‐α and IFN‐γ crucial for the regulation of asexual blood stage parasites.^12^ The Th1 immune response is accompanied by Th2 immune response, linked with production of IgG antibodies, which interfere with growth and advancement of asexual stages. In cases of coinfections, there is modulation in the naturally developed immune response against *P. falciparum* ^11^, ^13^ suggesting that coinfection with multiple parasites can compromise the host's naturally acquired immune response to a single parasite species and increase vulnerability to clinical illness.^14^, ^15^

Many strategies have been implemented to control and eradicate hookworm infection because of high morbidity associated with the infection.^16^ In widespread areas, hookworm monitoring depends on the regular mass administration with albendazole and other anthelmintic medications.^17^ However, most of these drugs fail to eliminate hookworm infection due to frequent re‐infection and evolving resistance to albendazole.^18^, ^19^ The current research strategy for human hookworm infection is vaccine development. Substantial improvement has been made in the production of anti‐hookworm vaccines, and current medical trials are investigating vaccines that target the infective L3 stage larvae and the adult worms.^18^ Evaluating immune response to hookworm L3 stage larvae could help provide explanations to the suboptimal effectiveness observed.^2^

We have previously established that *N. americanus* and *P. falciparum* coinfection increases IgG response to GMZ2 malaria vaccine candidate than those with only *P. falciparum* infection and that anthelmintic management of malaria‐hookworm coinfected individuals resulted in significant decrease in antibody responses against GMZ2 malaria vaccine candidate and constituent antigens.^13^ However, little is known about how *P. falciparum* coinfection affects antibody responses against hookworm L3 stage larvae in endemic areas. Moreover, studies assessing the effects *P. falciparum* coinfection with single species of hookworm are required to reduce masking effects of other species.^20^

Since malaria and hookworm infections often coincide geographically and their coinfections elicit complex immunomodulatory effects,^11^ we seek to ascertain the effect, the exposure of *P. falciparum* could have on naturally developed immune response against hookworm L3 stage larvae. Against this background, we evaluated the effect of *P. falciparum* infection on naturally acquired immunity against hookworm L3 stage larvae and how anthelmintic treatment affects immune response against Hookworm infection.

## 2 MATERIALS AND METHODS

### 2.1 Ethical approval and consent to participate

The Noguchi Memorial Institute for Medical Research Ethical Review Committee gave ethical approval for this study (FWA#: 00001824). All techniques employed were carried out in accordance with relevant guidelines and regulations.

### 2.2 Study site

The Kintampo North Municipality (KNM), in the Bono region of Ghana was the study site. KNM is in the forest‐savannah intermediate ecological section of middle Ghana. The KNM has a total area of 7162 km^2^ with a population of about 140,000 in 32,329 households. The residents are mostly subsistent farmers of crop and livestock.

### 2.3 Study design and sample processing

This longitudinal cross‐sectional study comprised collection of baseline sample and a follow‐up 3 weeks after‐anthelmintic treatment. Community engagement was done in each of the selected communities to explain the purpose and nature of the study. A total of 188 prospective study subjects, aged 4–88 years were randomly selected from a population census database. One hundred and eighty –three (*n* = 183) apparent healthy and consenting subjects were recruited for screening. Stool and blood samples were obtained from each participant.^21^, ^22^

### 2.4 Schematic representation of subject recruitment and selection

Skilled field workers administered structured health and demographic questionnaire to each participant. In addition, labeled stool‐collection containers was also shared to the participants. The following day, stool samples were collected for hookworm detection and finger pricks to prepare thin and thick blood film slides for malaria using rapid diagnostic test (RDT) kits (Abbott Diagnostics Korea Inc.) and light microscopy (Olympus) were used to detect malaria parasitemia and *P. falciparum*‐specific 18S rRNA gene using PCR.^21^ Stool hookworm infection was detected by the Kato‐Katz method and PCR was done for detecting stool hookworm and for speciation.^21^ Approximately 5 ml of venous blood was collected by venipuncture into hemogard SST® tubes (Becton Dickson and Company) from study participants. The blood sample was centrifuged (Lab centrifuge) and serum obtained was used for the immunological assays. Each participant was treated for hookworm infection with a single dose of albendazole (400 mg) and 3 weeks (21 days) after treatment.

### 2.5 Crude *N. americanus* L3 stage antigen preparation

L3 stage larvae of *N. americanus* were obtained from coproculture method using the Baerman techniques.^23^ The larvae were then suspended in 4°C 1X PBS at a concentration of about 500 larvae/ml. The larvae were homogenized on ice using prechilled high‐pressure homogenizer (Shanghai Nancheng Machinery Co., Ltd.). The solution was boiled, frozen in liquid nitrogen and thawed and homogenized three times. When approximately about 95% (or more) of the larvae were shredded/disrupted, the crude mixture was centrifuged at 4°C at 15,0000 rpm for 60 min using Lab centrifuge. The supernatant was collected and sterilized by passing it through a 0.2 µm filter. They were then aliquoted into 2 ml cryoval tubes and stored at −80°C until ready to be used for the ELISA analysis.

### 2.6 *N. americanus* antibody measurement in sera

Serum antibodies to *N. americanus* L3 stage antigen were measured using a modified version of a quantitative ELISA.^24^, ^25^ Briefly, ELISA plates (Nunc, Maxisorp, Fisher Scientific) were coated overnight at 4°C with 3.0µg/ml (100 µl/ml) of *N. americanus* L3 larva stage antigen diluted in PBS buffer (137 mM NaCl, 2.7 mM KCl, 8.1 mM Na_2_HPO_4_, KH_2_PO_4_, pH 7.2–7.4). The plates were washed 4 times with washing buffer (phosphate buffered saline [PBS]/0.1% Tween‐20; pH 7.2–7.4) and were then blocked for 1 h at room temperature (RT) with 200 µl of blocking buffer (PBS/0.1% Tween‐20/5% bovine serum albumin). The plates were tapped on a pad, and washed four times in washing buffer (For each washing step, the plates were filled with washing buffer for 1 min before they were emptied). Individual and positive control serum samples were diluted in PBS/0.1% Tween‐20/2.5% bovine serum albumin buffer (1:200 for IgG, IgA, and IgM and; 1:50 for IgG subclasses and IgE), 100 µl were added in duplicate to the respective wells, and plates were incubated for 2 h at RT. A pool of hyperimmune serum samples was twofold titrated downward with a starting dilution of 1:50 and 100 µl were added to each plate as a standard and also PBS buffer blank (serum dilution buffer) were added in duplicate to the wells. The plates were washed (4x) and 100 µl per well of alkaline phosphatase conjugated detection antibody in PBS/0.1% Tween‐20/2.5% bovine serum albumin buffer was added at the following dilutions: 1:3000 for IgG (Life technologies, Cat#. H10007) and IgA (Thermo Scientific, Cat #. PA 1‐74396), 1:1000 for IgG1, IgG2, IgG3, and IgG4 (The Binding Site), IgM 1: 5000 (Invitrogen); and IgE 1:1000 (Life technologies, Cat#. H15707) and incubated for 1 h at RT. The plates were washed, and treated with TMB (3,3′,5,5′‐tetramethylbenzidine) substrate (Kem‐En‐Tec Diagnosis A/S) and incubated at the following; 25 min for IgG and IgA, 10 min for IgM, and 30 min for IgG subclasses and IgE at RT in the dark until the color reaction were stopped with 0.2 M sulfuric acid (H_2_SO_4_). Optical density (OD) values were read at 450 nm with a reference wavelength of 570 nm with an ELISA reader (BioTek 405). The OD values obtained were converted into arbitrary antibody units (AU) using the four‐parameter curve fitting software (ADAMSEL, version 1.1 build 40 © 2009 EJ Remarque). To avoid differences that may have been due to inter‐plate variations, a two‐time point samples for each individual were tested on the same ELISA plate

### 2.7 Statistical analyses

Data analysis was performed using GraphPad Prism Version 8.0. Continuous variables were presented as means and standard deviations whilst categorical variables were presented as frequencies and percentages. Arbitrary antibody units were transformed into Log10 units. Linear regression was used to determine the association between antibody level and age. Association between antibody levels and infection status were assessed by multivariate linear regression analysis adjusting for age and sex with the endemic. The paired sample *t*‐test was used to assess if there was any significant difference in antibody levels before and after albendazole treatment. *p*‐values < 0.05 were considered statistically significant.

## 3 RESULTS

In the statistical analyses, we included 40 participants who were infected with *N. americanus* only, 63 participants coinfected with *N. americanus* and *P. falciparum* and 36 apparently healthy NEC. The *N. americanus* (31.88 ± 18.71 years) and NEC (36.33 ± 19.54 years) groups were significantly older than the *N. americanus*‐*P. falciparum* group (16.19 ± 10.55 years) (*p* < 0.0001). Also, gender was significantly associated with the participant's group (*p* = 0.0013). However, there was no significant difference in hookworm EPG among the study groups (*p* = 0.9950) (Table 1).

**Table 1:** Demographic and baseline characteristics of study participants

Study groups	Na (*n* = 40)	Na‐Pf (*n* = 63)	NEC (*n* = 36)	*p*‐value
**Gender [*n* (%)]**				0.0013^a^
Male	17 (42.5)	43 (68.3)	16 (44.4)	
Female	23 (57.5)	20 (31.7)	20 (55.6)	
Age (Mean ± SD)	31.88 ± 18.71	16.19 ± 10.55	36.33 ± 19.54	<0.0001^b^
Hookworm EPG (Median, IQR)	1092 (288, 2880)	1152 (576, 2304)	‐	0.9950^c^

Abbreviations: EPG, egg per gram; IQR, interquartile range; Na, *Necator americanus*; NEC, nonendemic controls; Pf, *Plasmodium falciparum*.

^a^ Chi‐square test.

^b^ Independent Sample *t*‐test.

^c^ Mann–Whitney *U* test.

### 3.1 Association between antihookworm L3 stage antibody levels and age

The association between hookworm L3 specific antibodies and age were assessed by linear regression before treatment. Increasing age was significantly associated with an increase in IgA (*r* ^2^ = 0.18, *p* < 0.0001) and IgG2 (*r* ^2^ = 0.10, *p* = 0.0017). However, increasing age was not significantly associated with IgE, IgG1, IgG3, IgG4, and IgM (*p* > 0.05)

### 3.2 Antibody responses against hookworm L3 stage antigens among study groups

Before treatment, the levels of antibody responses against hookworm L3 stage larvae were compared among individuals with different infection statuses. After adjusting for age and sex in a multiple linear regression model, individuals with *N. americanus* and *N. americanus*‐*P. falciparum* coinfection had generally higher levels of the five antibody isotypes, and IgG subclass antibodies compared to the NEC. Specifically, the *N. americanus* group had significantly higher levels of IgG3 (*β* = 0.39, 95% CI = [0.14–0.62], *p* = 0.002) compared to the control group. The *N. americanus*‐*P. falciparum* coinfected individuals also had significantly higher levels of IgE (*β* = 0.55, 95% CI = [0.70–0.39], *p* = 0.013) and IgG3 (*β* = 0.49, 95% CI = [0.22–0.76], *p* < 0.001) to hookworm L3 stage antigens compared to the NEC group. We found no significant difference in the IgA, IgG1, IgG2, IgG4, and IgM levels between the *P. falciparum* and NEC group (*p* > 0.05) or between the *N. americanus*‐*P. falciparum* and the NEC group (*p* > 0.05) (Table 2a).

**Table 2a:** Antibody responses against hookworm L3 stage antigen in hookworm infected and hookworm‐malaria coinfected individuals relative to the endemic controls

	*Na* only		*Na‐Pf* (coinfected)	
Antibody	β (95% CI)	*p*‐value	β (95% CI)	*p*‐value
IgA	0.10 (−0.05, 0.25)	0.193	0.02 (−0.13, 0.16)	0.842
IgE	0.17 (−0.03, 0.36)	0.090	0.55 (0.70, 0.39)	**0.013**
IgG1	0.06 (−0.08, 0.19)	0.407	0.04 (−0.12, 0.20)	0.625
IgG2	0.12 (−0.09, 0.33)	0.232	−0.01 (−0.23, 0.22)	0.948
IgG3	0.38 (0.14, 0.62)	0.002	0.49 (0.22, 0.76)	<0.001
IgG4	−0.16 (−0.45, 0.13)	0.277	−0.14 (−0.41, 0.13)	0.303
IgM	0.12 (−0.01, 0.24)	0.079	0.12 (−0.03, 0.26)	0.117

*Note*: Multivariate regression analysis adjusting for age and sex. β, estimated effect of covariate on antibody level; CI, confidence interval; *Na*, *Necator americanus*; *Pf, Plasmodium falciparum*. L3, hookworm larvae. Arbitrary antibody units were log_10_ transformed. EC group was set as reference for the model.

Furthermore, after adjusting for age and sex, malaria‐hookworm (*N. americanus*‐*P. falciparum*) coinfected participants had significantly higher levels of IgE (*β* = 0.30, 95% CI = [0.12, 0.48], *p* = 0.023] and IgG3 (*β* = 0.15, 95% CI = [0.02, 052], *p* = 0.004) compared to those infected with hookworm only (*N. americanus*). However, there was no significant difference in the IgA, IgG1, IgG2, IgG4, and IgM levels between the *N. americanus*‐*P. falciparum* coinfected group and the *N. americanus* group (*p* > 0.05) (Table 2b).

**Table 2b:** Antibody responses against hookworm L3 stage antigen among *Na/Pf* relative to hookworm infected participants

Antibody	*Na‐Pf* (coinfected)			
	Crude		Adjusted	
Isotypes	**β (95% CI)**	***p*‐value**	**β (95% CI)**	***p*‐value**
IgA	−0.18 (−0.30, −0.05)	0.007	−0.07 (−0.20, 0.07)	0.319
IgE	0.26 (0.15, 0.37)	0.003	0.30 (0.12, 0.48)	**0.023**
IgG1	−0.002 (−0.12, 0.12)	0.971	0.02 (−0.11, 0.16)	0.752
IgG2	−0.20 (−0.38, −0.03)	0.026	−0.09 (−0.28, 0.11)	0.367
IgG3	0.12 (0.02, 0.21)	0.013	0.27 (0.02, 052)	0.004
IgG4	0.07 (−0.20, 0.33)	0.624	0.11 (−0.19, 0.41)	0.459
IgM	−0.04 (−0.14, 0.06)	0.459	−0.03 (−0.14, 0.09)	0.635

*Note*: Multivariate regression analysis adjusting for age and sex. β, estimated effect of covariate on antibody level; CI, confidence interval; *Na*, *Necator americanus*; *Pf, Plasmodium falciparum*. L3, hookworm larvae. Arbitrary antibody units were log_10_ transformed. Hookworm only infected group was set as reference for the model.

### 3.3 Effect of albendazole treatment on antibody levels against hookworm L3 stage antigen

**Figure 1:**
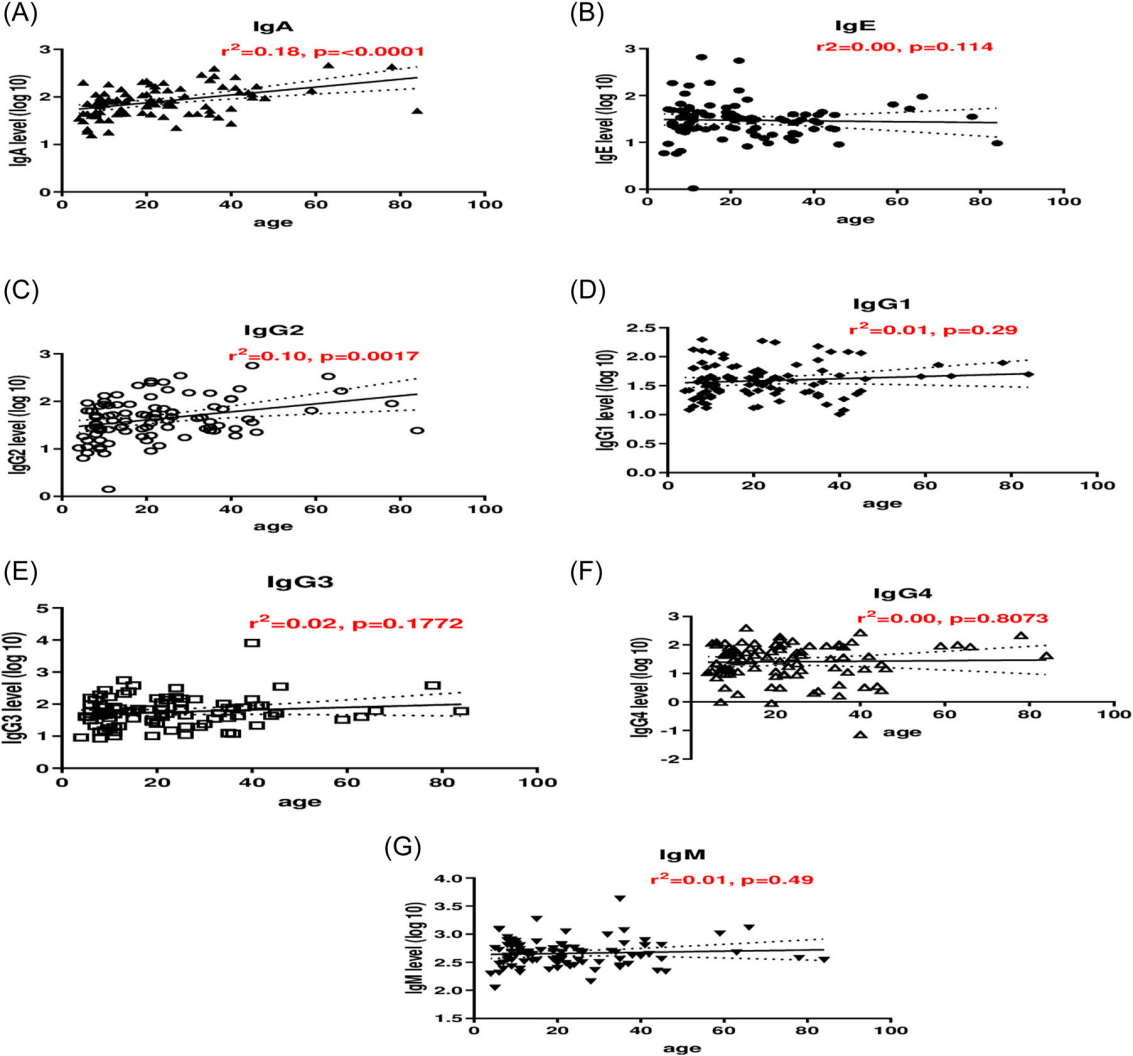
Isotype IgA, IgE, IgM, and IgG subclass levels in relation to age. Plots are shown (from top to bottom) for IgA (A), IgE (B), IgG2 (C), IgG1 (D), IgG3 (E), IgG4 (F), and IgM (G) levels against age (years). Analysis was done for individuals who were single infected with hookworm or coinfected with *Plasmodium falciparum* for the pretreatment data.

**Figure 2:**
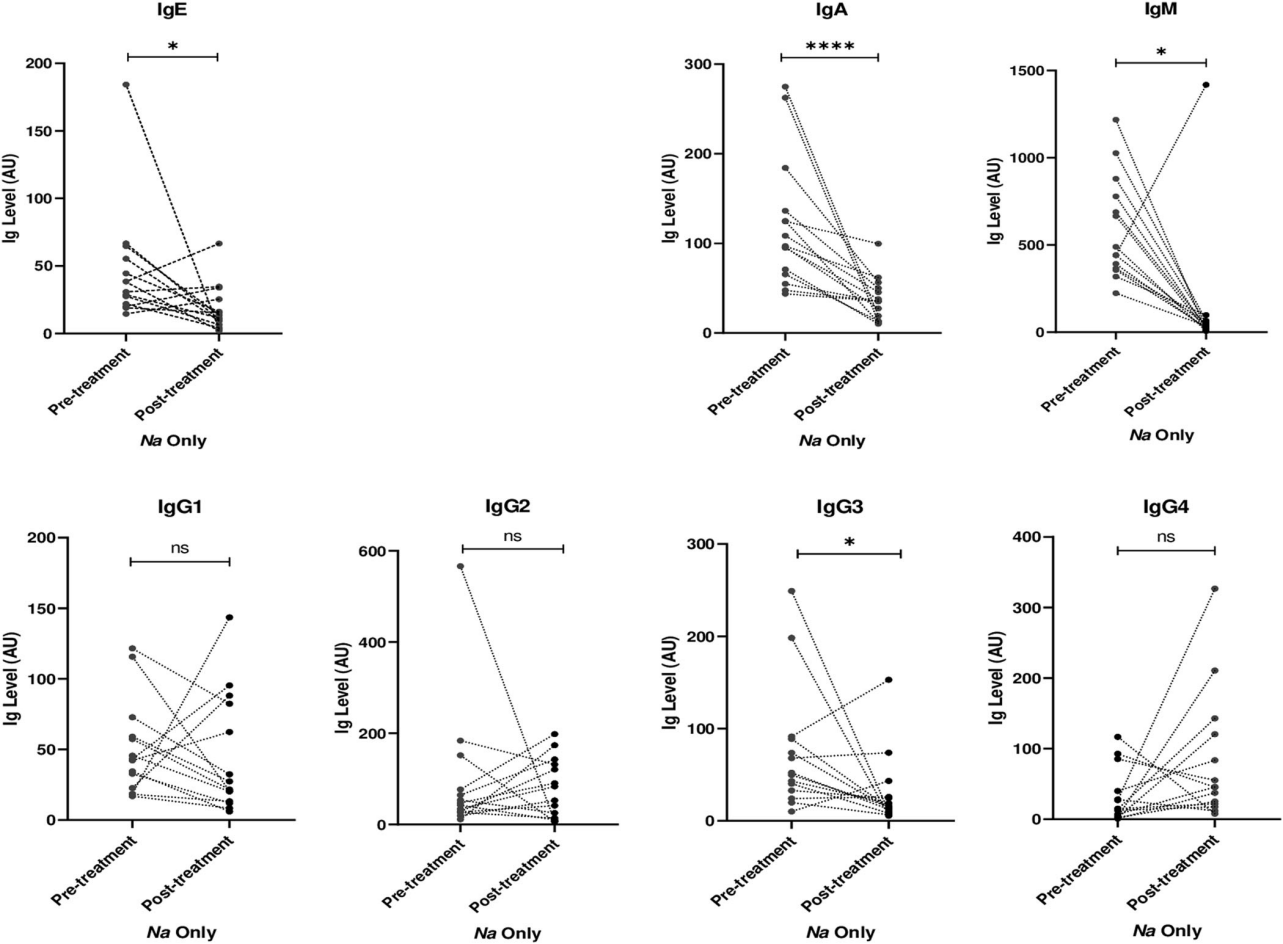
Antibody levels against hookworm L3 stage antigen in hookworm only infected individuals before and after albendazole treatment. *p*‐values computed by the paired sample *t*‐test after antibody titers were log transformed. * and **** represents *p* < 0.05 and *p* < 0.0001 respectively.

**Figure 3:**
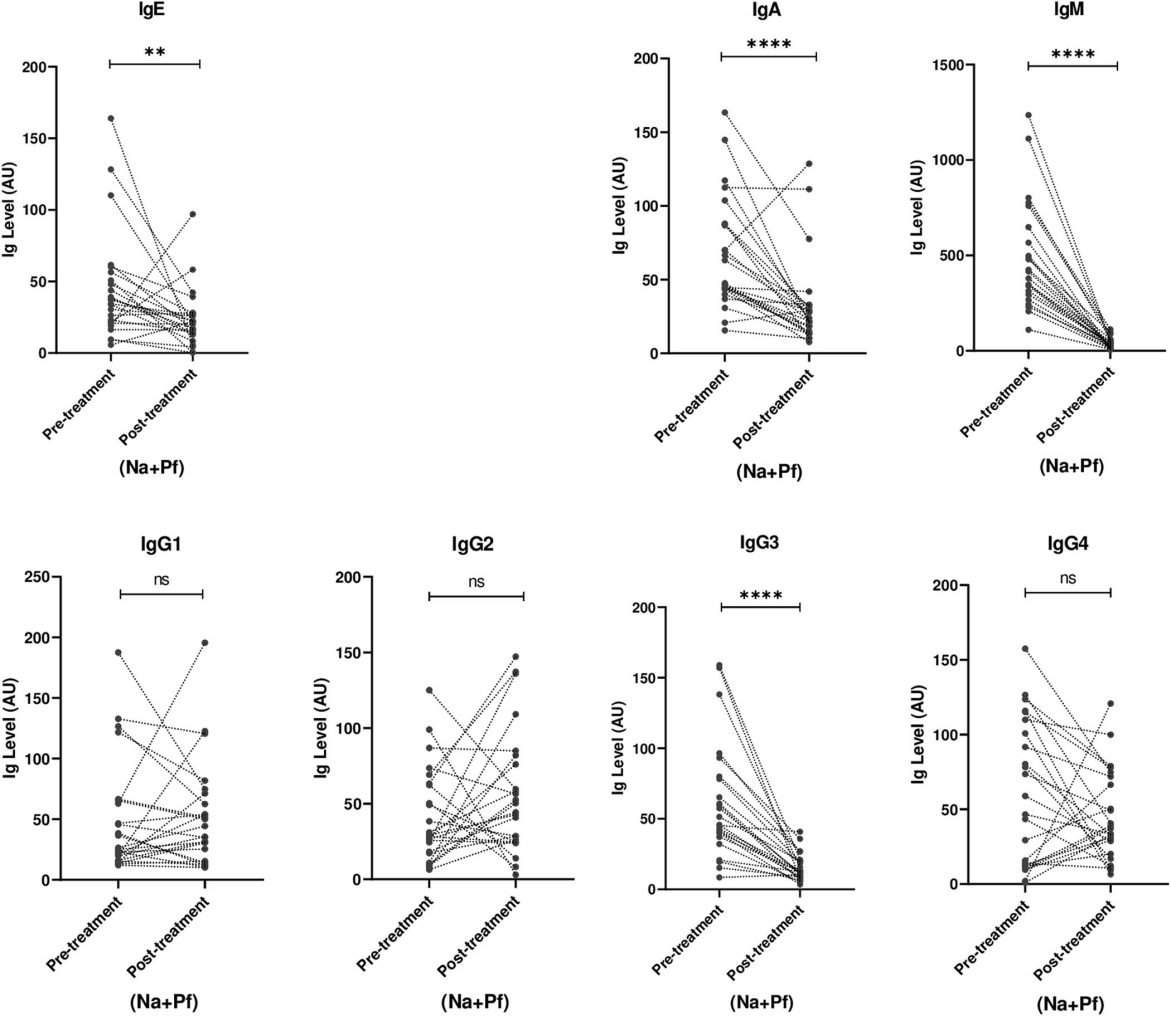
Antibody levels against hookworm L3 stage antigen in hookworm (*Necator americanus*) and *Plasmodium falciparum* coinfected (*N. americanus* and *P. falciparum*) individuals before and after albendazole treatment. *p*‐Values computed by the paired sample *t*‐test after antibody titers were log transformed. * and **** represents *p* < 0.01 and *p* < 0.0001 respectively.

Among *N. americanus* infected participants, albendazole treatment led to a significant reduction in IgE, IgA, IgM, and IgG3 antibodies against hookworm L3 stage larvae (*p* < 0.05) (Figures 1 and 2). Similarly, treating the *N. americanus*‐*P. falciparum* coinfected patients with albendazole resulted in significant reduction in IgE, IgA, IgM, and IgG3 antibodies against hookworm L3 stage larvae (*p* < 0.05) (Figure 3). However, among *N. americanus* and *N. americanus*‐*P. falciparum* coinfected individuals, albendazole treatment resulted in no significant change IgG1, IgG2, and IgG4 antibody responses against hookworm L3 stage larvae (*p* > 0.05) (Figures 2 and 3).

## 4 DISCUSSION

Hookworm (*N. americanus*) infection is widespread in sub‐Saharan Africa with prevalence of about 29% and affecting over 200 million people.^1^, ^26^, ^27^ Since malaria (*P. falciparum*) and hookworm (*N. americanus*) infections often coincide geographically and their coinfections elicit complex immunomodulatory effects,^11^ it is imperative to evaluate how their immune response interact. We have previously established that *N. americanus*‐*P. falciparum* coinfection increases IgG response to GMZ2 malaria vaccine candidate in the coinfected than those with only *P. falciparum* infection but albendazole treatment of hookworm‐malaria coinfected individuals resulted in significant reduction in antibody responses against GMZ2 malaria vaccine candidate and constituent antigens.^13^ Currently, little is known about how *P. falciparum* coinfection affects antibody responses against hookworm L3 stage larvae in endemic areas. Moreover, studies assessing the effects of *P. falciparum* coinfection with single species of hookworm is required to reduce masking effects of other species.^20^ In the current study, we hypothesized that *P. falciparum* coinfection and anthelminthic treatment would have effect on naturally acquired immune response against hookworm L3 stage larvae. We observed that *P. falciparum* coinfection is associated with improved IgE and IgG response against hookworm L3 stage larvae. However, single dose of albendazole treatment led to significant reduction in naturally acquired immune response against hookworm infection.

The consequence of *P. falciparum* infection on naturally acquired antibody response against helminth infection has always involved a pool of different worm species into one single variable and the few studies had focused on *Schistosoma haematobium* species.^28^, ^29^ In the present study, *P. falciparum* coinfection was associated with improved IgE and IgG3 response against hookworm L3 stage larvae. The present study findings is similar with a study by Remoue et al.,^30^ who reported significantly higher IgG3 and IgE responses to *S. haematobium antigens* in coinfected children with *P. falciparum* compared to children with *S. haematobium* monoinfection. In another study, Imai et al.^31^ found higher IgE response against *S. haematobium* antigens among young children who are concurrently exposed to *P. falciparum* and *S. haematobium*. The higher antibody response found in the *P. falciparum‐N. americanus* coinfected individuals than the *N. americanus* monoinfected infected individuals could be partly explained by the ability of *P. falciparum* and *N. americanus* to prompt polyclonal B cell responses,^32^, ^33^ which are predominantly apparent for IgE during hookworm infections.^9^ This mechanism includes the stimulation and distinction of antibody‐secreting cells from different B cell clones, irrespective of their antigen specificity. Despite the fact this can play an essential role in protection against infections by augmenting normal antibody production, it can also be a parasitic plan to avoid host‐specific immune made available under responses by neutralizing antibodies against essential epitopes for protection.^34^

Studies have highlighted the protective role of malaria‐helminth coinfection. In recent studies, Tokplonou et al.^35^ and Amoani et al.^13^ demonstrated that hookworm‐malaria coinfected individuals produced higher antibody response against malaria GMZ and GLURP candidate vaccine antigens compared to monoinfected individuals. Moreover, Diallo et al.^36^ showed that Schistosomiasis coinfection in children enhances acquired immune response against *P. falciparum* malaria antigens. These findings had led to recent hypothesis that malaria and hookworm coinfection may pose synergistic effect at the humoral immune system.^37^, ^38^ This may be explained by the fact that antibody production requires Th‐2 cytokines such as IL‐4,^39^, ^40^ and since the two parasites are able to induce Th‐2 responses, higher levels of antibodies could occur during the course of infection. Nevertheless, it is imperative that the protective ability of these antibodies are evaluated in by looking at the subclass composition of the IgG responses in a longitudinal study.

The current study further observed that single dose of albendazole treatment resulted in significant reduction in IgE, IgA, IgM, and IgG3 antibodies against hookworm L3 stage larvae in both *N. americanus* and *N. americanus*‐*P. falciparum* coinfected groups. Consistent with our study, Pinot de Moira et al.,^41^ reported a decrease in IgE, IgG1, and IgG4 response to *N. americanus* and *S. mansoni* eggs following albendazole and praziquantel teratment. However, they recorded a significant increase in antibody response against adult worms after treatment. Among *P. falciparum*‐hookworm coinfected individuals, albendazole treatment resulted in significant reduction IgG1, IgG3, and IgM response against GMZ malaria vaccine.^13^ A possible mechanism explaining the reduction in immune response following albendazole treatment is the fact that *P. falciparum*‐hookworm infections are associated with Th2 immune response^42^, ^43^ and subsequent increased levels of IL‐10, provide help for B cells to produce antibodies.^21^, ^22^ Given the effect of albendazole treatment on IgE, IgA, IgM, and IgG3 antibodies against hookworm L3 stage larvae, it would appear that deworming decreases Th2‐mediated responses. The major influence seen on antibody levels 3 weeks after albendazole treatment probably reflects the decrease in prevalence of active hookworm infections. The findings of this study explained the impacts of *P. falciparum* coinfection on naturally acquired immune response against hookworm infection suggesting that malaria coinfection may have a beneficial role in hookworm vaccination. However, further studies are required to explore whether the higher antibody responses are protective. It is imperative to confirm in a longitudinal study whether the higher antibody responses in the coinfected individuals are protective against hookworm infection.

The study had few limitations. The study was conducted with relatively low sample size with unmatched groups. However, age and sex of participants were adjusted in multivariate analyses. Additionally, the study could not determine causativeness because of the study design employed (cross‐sectional). A longitudinal design to determine the susceptibility of *N. americanus*‐*P. falciparum* coinfected participants to subsequent infections would be more appropriate.

## 5 CONCLUSION

*P. falciparum* coinfection is associated with improved IgE and IgG response against hookworm L3 stage larvae. Single‐dose treatment with albendazole led to significant reduction in naturally acquired immune response against hookworm infection. Thus, *P. falciparum* infection can have a boosting effect on hookworm vaccine efficacy.

## AUTHOR CONTRIBUTIONS

**Samuel A. Sakyi**: Conceptualization; data curation; formal analysis; methodology; resources; writing—original draft; writing—review and editing. **Michael D. Wilson**: Conceptualization; formal analysis; funding acquisition; project administration; resources; supervision; visualization. **Bright Adu**: Conceptualization; data curation; funding acquisition; investigation; methodology; software; writing—original draft; writing—review and editing. **Stephen Opoku**: Formal analysis; investigation; methodology; resources; software; writing—original draft; writing—review and editing. **Antwi Brewoo**: Data curation; investigation; methodology; project administration; writing—original draft; writing—review and editing. **Amma Larbi**: Conceptualization; formal analysis; investigation; methodology; project administration; validation; writing—original draft; writing—review and editing. **Samuel K. Tchum**: Investigation; methodology; project administration; resources; software; supervision; writing—original draft; writing—review and editing. **Roland O. Saahene**: Conceptualization; formal analysis; funding acquisition; investigation; methodology; project administration; writing—review and editing. **Wilfred Aniagyei**: Conceptualization; software; validation; visualization; writing—original draft; writing—review and editing. **Christian Sewor**: Data curation; investigation; methodology; project administration; writing—original draft; writing—review and editing. **David Courtin**: Project administration; resources; software; supervision; validation; writing—review and editing. **Michael Cappello**: Resources; software; supervision; validation; visualization; writing—original draft; writing—review and editing. **Ben Gyan**: Conceptualization; formal analysis; project administration; resources; software; supervision. **Benjamin Amoani**: Conceptualization; data curation; formal analysis; investigation; methodology; writing—original draft; writing—review and editing.

## CONFLICT OF INTEREST

The authors declare no conflict of interest.

## TRANSPARANCY STATEMENT

The author affirms that this manuscript is an honest, accurate and transparent account of the study have not been omitted.

## Data Availability

All data generated or analyzed during this study are included in this article and its supplementary information files data and can be requested from corresponding author.
